# Glomerular Yield in Ultrasound-Guided Native Kidney Biopsies in Pediatric and Adult Populations With Age-Stratified Subgroups

**DOI:** 10.7759/cureus.91202

**Published:** 2025-08-28

**Authors:** Pedro C Ravizzini, Henrique A Lino, Eduardo de Faria Castro Fleury, Luis Gustavo M Toledo

**Affiliations:** 1 Department of Radiology, Faculty of Medical Sciences of Santa Casa de Misericordia de São Paulo, São Paulo, BRA; 2 Department of Urology, Faculty of Medical Sciences of Santa Casa de Misericordia de São Paulo, São Paulo, BRA

**Keywords:** adult, biopsy cores, children, complications, glomerular density, glomerular yield, native kidney, pediatric, percutaneous, ultrasound-guided biopsy

## Abstract

Background: This study compares the glomerular yield, mean number of biopsy cores, and complication rates between pediatric and adult populations undergoing ultrasound-guided native kidney biopsies. Additionally, the adult population was stratified into subgroups to further analyze age-related differences in glomerular yield and complication rates.

Materials and methods: A retrospective cohort study was conducted using data from 431 native kidney biopsies performed between February 2008 and February 2021. Patients were divided into two main groups: pediatric (≤18 years old, n = 215) and adult (>18 years old, n = 216). Adult patients were further stratified into three subgroups: those aged >18 to 40 years old (n = 108), >40 to 60 years old (n = 72), and >60 years old (n = 36).

Results: Pediatric patients had a higher mean number of glomeruli per core than adults (20.6 ± 12.3 vs. 15.7 ± 9.4; p < 0.001). Among adults, yield declined with age: 7.2 ± 9.8 (>18-40 years), 15.1 ± 9.1 (>40-60 years), and 13.8 ± 8.7 (>60 years) (p < 0.05). No difference in core numbers between pediatric and adults (2.4 ± 0.66 vs. 2.5 ± 0.66; p = 0.08) or among adult subgroups was observed. In the adult stratification group, the >18 to 40-year-old subgroup had the highest mean number of glomeruli (17.2 ± 9.8), followed by the >40 to 60-year-old (15.1 ± 9.1) and >60-year-old subgroups (13.8 ± 8.7) (p<0.05). Complication rates were low across all groups, with no significant differences observed between the pediatric and adult populations (12/215, 5.6% vs. 8/216, 3.7%; p = 0.454). Among the adult subgroups, the >18 to 40-year-old subgroup had the lowest complication rate (3/108, 2.8%), followed by the >40 to 60-year-old (3/72, 4.2%) and >60-year-old subgroups (2/36, 5.6%). However, these differences were not statistically significant (p = 0.512).

Conclusions: Pediatric patients exhibit a higher glomerular yield per biopsy core compared to adults, with no significant difference in the number of biopsy cores obtained. Among adults, younger patients (>18 to 40 years) demonstrate a higher glomerular yield compared to older age groups, suggesting that age may influence glomerular density and biopsy outcomes. Complication rates were low across all age groups, with no significant differences observed. These findings highlight the importance of considering age-related factors when performing native kidney biopsies to optimize diagnostic yield and minimize complications.

## Introduction

Native kidney biopsy is a cornerstone diagnostic procedure in nephrology, providing histopathological insights that inform diagnosis, prognosis, and treatment strategies for a wide range of renal diseases [1-3]. Since its introduction, the technique has undergone significant evolution, with ultrasound-guided percutaneous biopsy emerging as the gold standard due to its safety, efficacy, and ability to yield adequate renal tissue for analysis [4,5].

One of the most important determinants of a successful kidney biopsy is the glomerular yield, defined as the number of glomeruli obtained in the biopsy samples. Adequate glomerular yield is essential for accurate histopathological diagnosis, with a minimum of 10 glomeruli per sample considered optimal for reliable interpretation [6].

The diagnostic utility of kidney biopsies, however, is influenced by several factors, including patient age, renal anatomy, and comorbid conditions. While previous studies have compared biopsy outcomes between pediatric and adult populations, there is limited research on how age-related factors within the adult population influence glomerular yield and complication rates. Age-related changes in renal structure, such as glomerulosclerosis, interstitial fibrosis, and reduced glomerular density, are well-documented and may impact biopsy outcomes [7,8]. Additionally, the prevalence of comorbid conditions like hypertension and diabetes, which are more common in older adults, may further complicate biopsy procedures and outcomes [9].

This study aims to address existing gaps by comparing the mean number of biopsy cores, glomeruli per core, and complication rates between pediatric populations (≤18 years) and adult populations (>18 years). Additionally, it seeks to stratify the adult population into subgroups (>18 to 40, >40 to 60, and >60 years) to investigate how age-related variations in glomerular yield may influence diagnostic adequacy, thereby providing insights for optimizing biopsy protocols across different age groups.

## Materials and methods

This retrospective cohort study evaluated 431 consecutive ultrasound-guided native kidney biopsies performed at the Interventional Radiology Service of Santa Casa de Misericordia de São Paulo, Brazil, between February 2008 and February 2021. Patients were divided into two main groups: pediatric (≤18 years, n = 215) and adult (>18 years, n = 216). The adult group was further stratified into three age-based subgroups: >18-40 years (n = 108), >40-60 years (n = 72), and >60 years (n = 36). Exclusion criteria included biopsies of transplanted kidneys, incomplete procedural records, or insufficient tissue for histopathological analysis.

All biopsies were performed by a single interventional radiologist under real-time ultrasound guidance using an Envisor C HD or Affiniti 70 device (Philips Ultrasound Inc., Bothell, WA), with a 3.5-5 MHz convex transducer. Patients were positioned in the free-flank supine anterolateral decubitus, as previously described [10]. A semi-automatic 18-gauge needle (SuperCore II, Argon Medical, Frisco, TX) was used to obtain at least two cores per biopsy. Additional passes were performed if initial samples were deemed inadequate based on tissue length less than 1 cm.

For glomerular yield quantification, biopsy cores were fixed in 10% formalin, embedded in paraffin, and sectioned at a thickness of 2-3 μm. Tissue sections were stained with hematoxylin and eosin (H&E), periodic acid-Schiff (PAS), and Masson’s trichrome. A renal pathologist blinded to the clinical data counted the number of intact glomeruli per core under light microscopy at ×200 magnification, excluding those that were globally sclerotic.

Primary outcomes included the mean number of glomeruli per core (total glomeruli divided by the number of cores) and the proportion of biopsies achieving the diagnostic threshold of ≥10 glomeruli [6]. A biopsy core was considered non-optimal if it measured less than 1.0 mm in length or contained fewer than ten glomeruli. Secondary outcomes were the mean number of biopsy cores per procedure and complication rates, categorized as major or minor as presented in Gesualdo et al. and Visconti et al. [2,11].

Statistical analysis was performed using SPSS Statistics version 25.0 (IBM Corp. Released 2017. IBM SPSS Statistics for Windows, Version 25.0. Armonk, NY: IBM Corp.). Continuous variables were expressed as mean ± standard deviation (SD), and categorical variables as frequencies. The Mann-Whitney U test was used to compare glomerular yield and core counts between pediatric and adult groups. Differences among adult age subgroups were assessed using ANOVA with post-hoc Tukey tests. Complication rates and diagnostic adequacy were analyzed using the chi-square test. A p-value <0.05 was considered statistically significant. The study was approved by the Institutional Review Board of Santa Casa de Misericordia de São Paulo (approval number: 48707221.4.0000.5479).

## Results

Demographic data are depicted in Table 1. The pediatric group had a significantly higher mean number of glomeruli per biopsy core compared to the adult group (Table 2); however, there was no significant difference in the mean number of biopsy cores between the pediatric and adult groups (2.4 ± 0.66 vs. 2.5 ± 0.66; p = 0.08). Overall complication rates were low, and no differences were observed between the children and adult groups (Table 3).

**Table 1:** Demographic characteristics of the study population * ANOVA was used for BMI comparison between pediatric and adult groups. ANOVA: analysis of variance, BMI: body mass index, SD: standard deviation, NA: not analyzed, N-A: not applicable

Age group (years)	n	Mean age (years ± SD)	Age range (years)	Mean BMI (kg/m² ± SD)	BMI range (kg/m²)	p-value (BMI)	Test statistic
Pediatric (≤18)	215	10.5 ± 4.2	1-18	18.3 ± 3.1	12.0-24.5	<0.001*	F = 15.7
Adult subgroups							
>18 to 40	108	29.7 ± 6.5	18.1-40	24.5 ± 4.2	16.8-35.2	NA	N-A
>40 to 60	72	50.3 ± 5.8	40.1-60	27.8 ± 5.1	18.6-38.4	NA	N-A
>60	36	68.4 ± 6.2	60.1-85	28.6 ± 4.9	20.5-39.7	NA	N-A

**Table 2:** Mean number of biopsy cores and glomeruli obtained per case * Mann-Whitney U test for pediatric vs. adult comparison, † ANOVA with post-hoc t-tests for adult subgroups SD: standard deviation, N-A: not applicable

Group	n	Range (glomeruli)	Mean glomeruli	SD	p-value	Test statistic
Pediatric (≤18)	215	4-66	20.6	12.3	<0.001*	U = 18,542
Adult (overall)	216	0-53	15.7	9.4	Reference	N-A
Adult subgroups						
>18 to 40 years	108	1-53	17.2	9.8	<0.05† (vs. >60)	F = 4.32
>40 to 60 years	72	0-44	15.1	9.1	0.08 (vs. >18-40)	t = 1.76
>60 years	36	0-37	13.8	8.7	0.03 (vs. >18-40)	t = 2.21

**Table 3:** Complication rates and inconclusive results by age group * Chi-square test for pediatric vs. adult comparisons, † Chi-square or Fisher’s exact test for adult subgroup comparisons N-A: not applicable

Age group (years)	n	Complication rate	p-value (complications)	Test statistic	Inconclusive results	p-value (inconclusive)	Test statistic
Pediatric (≤18)	215	12 (5.6%)	0.454*	χ² = 0.56	45 (20.9%)	0.021*	χ² = 5.32
Adult (>18)	216	8 (3.7%)	Reference	N-A	38 (17.6%)	Reference	Not applicable
Adult subgroups							
>18 to 40	108	3 (2.8%)	0.823† (vs. >40-60)	χ² = 0.05	15 (13.9%)	0.038† (vs. >60)	χ² = 4.29
>40 to 60	72	3 (4.2%)	0.823† (vs. >60)	χ² = 0.05	12 (16.7%)	0.823† (vs. >18-40)	χ² = 0.05
>60	36	2 (5.6%)	0.454† (vs. >18-40)	χ² = 0.56	11 (30.6%)	0.003† (vs. >18-40)	χ² = 8.76

The mean number of biopsy cores also did not differ significantly when comparing the adult age-stratified subgroups (p = 0.12). Among the subgroups, the >18 to 40-year-old subgroup had the highest number of glomeruli per core, followed by the >40 to 60-year-old and the >60-year-old subgroups (Table 2). When comparing the mean number of glomeruli in the >18 to 40-year-old and >40 to 60-year-old subgroups and the >40 to 60-year-old and >60-year-old subgroups, no statistical significance was found (Table 2). The >60-year-old subgroup had significantly fewer glomeruli per core compared to the >18 to 40-year-old subgroup (Table 2). Complication rates and the inconclusive histopathological results are shown in Table 3.

The proportion of biopsy samples containing at least 10 glomeruli was higher in the pediatric group compared to the adult group (164/215, 76.3% vs. 148/216, 68.5%; p = 0.072). Among the adult subgroups, the >18 to 40-year-old subgroup had the highest proportion of samples with ≥10 glomeruli (78/108, 72.2%), followed by the >40 to 60-year-old subgroup (48/72, 66.7%) and the >60-year-old subgroup (22/36, 61.1%).

In the pediatric group (n = 215), the most prevalent conditions were focal segmental glomerulosclerosis (69/215, 32.1%), followed by minimal histological lesion (55/215, 25.6%) and proliferative mesangial glomerulonephritis (39/215, 18.3%). In the >18 to 40-year-old subgroup (n = 108), segmental and focal glomerulosclerosis was the most common diagnosis (31/108, 28.7%), followed by membranous glomerulonephritis (24/108, 22.4%) and proliferative mesangial glomerulonephritis (21/108, 19.8%). In the >40 to 60-year-old subgroup (n = 72), segmental and focal glomerulosclerosis remained the leading condition (22/72, 30.2%), followed by membranous glomerulonephritis (18/72, 25.6%) and diabetic nephrosclerosis (14/72, 18.9%). In the >60-year-old subgroup (n = 36), segmental and focal glomerulosclerosis was again the most frequent diagnosis (13/36, 35.4%), while diabetic nephrosclerosis (10/36, 28.3%) and membranous glomerulonephritis (7/36, 20.1%) were also observed. Segmental and focal glomerulosclerosis consistently emerged as a predominant condition in all cohorts.

## Discussion

This study demonstrates that pediatric patients exhibit a higher glomerular yield per biopsy core compared to adults (Table 2), which may be attributed to differences in glomerular density and renal parenchymal characteristics between these populations. The lack of a significant difference in the number of biopsy cores suggests that the higher glomerular yield in pediatric patients is not due to a greater number of cores but rather to the quality of the tissue obtained. These results align with studies showing that younger patients have higher glomerular density, which declines with age due to factors such as hypertension, diabetes, and other age-related renal changes [7,12].

Adequate glomerular yield is essential for adequate interpretation of biopsy samples, with a minimum of 10 glomeruli per sample generally considered optimal [6]. However, achieving this yield can be challenging, particularly in older adults or patients with advanced renal disease, where glomerulosclerosis and interstitial fibrosis are more prevalent [7,8]. Additionally, certain conditions may result in fewer intact glomeruli being available for sampling. For instance, diabetic nephrosclerosis or advanced glomerulosclerosis may result in lower glomerular counts, complicating the diagnostic process [7,8].

Additional considerations can also be drawn regarding the technique and equipment used during the biopsy procedures. Typically, the same biopsy needle gauge (e.g., 18 G) is used for both pediatric and adult patients, which may not always be ideal. While 18-G needles are generally safe and effective, they may not yield sufficient tissue, particularly in older adults or patients with dense renal fibrosis. Conversely, larger-gauge needles (e.g., 16 G) may yield more glomeruli but could increase the risk of complications, especially in pediatric patients or those with fragile renal tissue [13]. This uniform approach to biopsy technique and needle selection may contribute to variations in complication rates and diagnostic yield across different age groups and disease types.

The primary goal of ultrasound-guided native kidney biopsies is to obtain adequate biopsy samples with minimal complication rates. However, this balance is crucial, as inadequate sampling can lead to inconclusive results, necessitating repeat biopsies and increasing patient risk. On the other hand, excessive tissue sampling or the use of larger-gauge needles may increase the likelihood of complications such as bleeding or hematoma, particularly in older adults or those with comorbid conditions like hypertension or diabetes [13,14]. Therefore, optimizing technique while minimizing risks remains a key focus in ultrasound-guided native kidney biopsies. This paper provides a comprehensive analysis of glomerular yield and complication rates in native kidney biopsies across pediatric and adult populations. The results obtained underscore the importance of considering age-related factors when performing kidney biopsies to optimize diagnostic yield and minimize complications.

The lack of difference in the number of biopsy cores between adult and pediatric groups suggests that the higher yield in pediatric patients is not due to a greater number of cores but instead to the higher glomerular density of the tissue obtained. In fact, fewer age-related structural changes in the kidneys of younger patients may favor a higher glomerular density [7,12]. This suggests that pediatric patients may require fewer cores to achieve an adequate diagnostic yield, potentially reducing procedural time and associated risks.

Table 2 further stratifies the adult population into age subgroups, revealing a progressive decline in glomerular yield with age. The mean number of glomeruli was slightly lower when comparing the >18 to 40-year-old subgroup to the >40 to 60-year-old subgroup, but the difference was not significant (p = 0.08). Between the >40 to 60-year-old subgroup and the >60-year-old subgroup, the difference was also not significant (p = 0.25), although the trend showed a further reduction in glomerular yield. The >60-year-old subgroup had significantly fewer glomeruli compared to the >18 to 40-year-old subgroup (p = 0.03). Those findings suggest an age-related gradual decline in glomeruli count with age, with the most pronounced difference occurring after 60 years. In fact, age-related changes in renal structure, such as glomerulosclerosis and interstitial fibrosis, have been demonstrated to contribute to lower glomerular density in older adults [7,8], coupled with a decline in the natural reduction in glomerular filtration rate that occurs with aging [9]. Therefore, younger adults (>18 to 40 years) may have more favorable biopsy outcomes and a higher glomerular yield, while older adults, particularly those over 60, may require additional considerations to optimize diagnostic yield.

Complication rates were low across all age groups, with no significant differences observed between pediatric and adult populations (Table 3). Among the adult subgroups, the >18 to 40-year-old subgroup had the lowest complication rate, followed by the >40 to 60-year-old subgroup and the >60-year-old subgroup (Table 3). Although these differences were not statistically significant, the slightly higher complication rate in the >60-year-old subgroup may be correlated with age-related vascular fragility and comorbid conditions, which are more prevalent in older adults [14]. Nevertheless, these findings suggest that percutaneous renal biopsies are generally safe when performed with real-time ultrasound guidance, even in older patients, as previously shown [13,15]. However, the slightly higher complication rate in older adults underscores the need for careful management of comorbid conditions to minimize risks.

Variations in inconclusive histopathological results across age groups are depicted in Table 3. The pediatric group had a slightly higher prevalence of inconclusive results compared to the overall adult population (Table 3). This may reflect technical challenges in obtaining adequate tissue samples in younger patients, despite their higher glomerular density. Among the adult subgroups, the >60-year-old subgroup had the highest prevalence of inconclusive results (11/36, 30.6%), which aligns with the decline in glomerular yield in older age groups but may also relate to age-related structural changes in the kidney, as well as comorbid conditions that compromise renal tissue integrity [7,8]. Hence, age-specific strategies could be implemented to optimize biopsy outcomes, particularly in older adults and pediatric patients, thereby reducing the likelihood of inconclusive results and improving diagnostic accuracy.

The findings in this study collectively highlight the importance of considering age-related factors when performing native kidney biopsies. Pediatric patients exhibit a higher glomerular yield per biopsy core, which may reduce the need for multiple cores. In contrast, older adults, particularly those over 60, may require additional considerations to optimize diagnostic yield and minimize complications. The use of larger-gauge needles (e.g., 16 G) may be beneficial in patients requiring a higher diagnostic yield, as suggested by previous studies [13]. However, in older adults or those at higher risk of complications, the use of 18-G needles may be more appropriate to balance diagnostic yield with safety [14]. Additionally, managing comorbid conditions such as hypertension and diabetes in older adults may improve biopsy outcomes by reducing the risk of inconclusive results and complications [13,14]. Nevertheless, advanced age alone should not deter clinicians from performing kidney biopsies in older adults, as the procedure remains both safe and effective, with adequate glomerular yield in elderly patients over 65 years [15].

This study has several important implications for clinical practice. First, the higher glomerular yield in pediatric patients suggests fewer biopsy cores may be needed to achieve an adequate diagnostic yield, potentially reducing procedural time and risks. For older adults, particularly those over 60, the lower glomerular yield underscores the importance of careful planning and potentially additional cores to ensure sufficient tissue for accurate diagnosis. Clinicians should also consider the type of kidney disease when performing biopsies, as certain conditions may affect glomerular density and yield.

However, this study has several limitations that should be acknowledged. First, it is a single-center, single-operator study, as the same interventional radiologist performed all biopsies. While this approach ensures consistency in technique, it may limit the generalizability of the findings, as operator experience and institutional protocols can influence biopsy outcomes. Future multi-center studies involving multiple operators would provide a more comprehensive understanding of how these factors impact glomerular yield and complication rates. While no statistically significant differences in complication rates were found between pediatric and adult populations, a slightly higher rate of complications was observed in children (5.6% vs. 3.7%). This finding may still be clinically relevant, particularly given the higher glomerular density in pediatric patients. It may be worth considering the use of smaller-gauge needles in children to reduce the risk of complications, as their higher glomerular density may allow for adequate diagnostic yield with fewer risks [13]. Although we adjusted for common comorbidities, the impact of specific renal pathologies (e.g., diabetic nephropathy or amyloidosis) on glomerular yield was not separately analyzed due to sample size constraints. Future studies could explore disease-specific effects in larger cohorts.

## Conclusions

This study demonstrates that pediatric patients exhibit a higher glomerular yield per biopsy core compared to adults. Biopsies in younger adults also demonstrated a higher glomerular yield compared to older age groups, suggesting that age may influence glomerular density and the outcomes of biopsies. These findings highlight the importance of considering age-related factors when performing native kidney biopsies to optimize diagnostic yield and minimize complications. Future studies should explore the impact of comorbid conditions, biopsy techniques, and needle size on biopsy outcomes, particularly in older adults and pediatric patients.
